# Comparison of the Structural, Cytological and Biomarker Expression in Carcinoma in situ and Invasive Components in Breast Carcinoma

**DOI:** 10.30699/ijp.2024.2025907.3285

**Published:** 2024-04-07

**Authors:** Azar Naimi, Niloufar Mohaghegh

**Affiliations:** 1 Department of Pathology, School of Medicine Al-Zahra Hospital, Isfahan University of Medical Sciences, Isfahan, Iran; 2 Department of Pathology, School of Medicine, Isfahan University of Medical Sciences, Isfahan, Iran

**Keywords:** in situ breast carcinoma, invasive breast carcinoma, nuclear grade, structural patterns, immunohistochemical markers

## Abstract

**Background & Objective:**

Breast cancer is thought to arise from non-invasive breast lesions, such as atypical ductal hyperplasia (ADH) and ductal carcinoma in situ (DCIS). DCIS is considered a direct precursor of invasive carcinoma. The morphological features alone do not reflect the biological truth of this disease. Therefore, we investigated features of carcinoma in situ and the invasive components in women diagnosed with breast cancer.

**Methods:**

This study was a cross-sectional study. The corresponding IHC slides were selected from the pathology archive and examined by the pathologist. Fifty-one samples which showed both in situ and invasive components confirmed immunohistochemically, were included in the study.

**Results:**

In 70.6% of the cases a high grade of in situ and invasive carcinoma was observed. In 45.1% of the studied cases, a solid structure was observed in in-situ carcinoma, and no otherwise specified structure was observed in invasive carcinoma. In 74.5% of both in situ and invasive carcinoma types, ER.PR had a positive value. In 45.5% of the cases, both in situ and invasive carcinoma components show low Ki67. In 42.2%, both in situ and invasive carcinomas were Her2 negative. There was no significant difference between the grade (*P*=0.687), Her2 type (*P*=0.532), and structure (*P*=0.532). ER.PR (*P*=1.00) and Ki67 (*P*=0.180) of in situ and invasive carcinoma in this study.

**Conclusion:**

Our study showed differences between in situ and invasive biomarker expression. According to our findings, owing to heterogeneity, in situ components can't be representative of invasive components for treatment choices.

## Introduction

Breast cancer is the most prevalent type of cancer among women. In recent years, thanks to advancements in early diagnosis and treatment methods, the death rate has decreased significantly. However, it is the second leading cause of cancer-related deaths in women in Europe and Western countries, following lung cancer (1). Breast cancer is also the most prevalent cancer among Iranian women. The prevalence of breast cancer among Iranian women has reached 22%. Breast cancer tends to occur at a lower age incidence in Iranian society (2). In some studies, it has been observed that the mean age of breast cancer onset is estimated to be 15 years lower in Iran compared to the global average (3). The cause of breast cancer is not fully understood, but it is believed that genetic factors and hormonal effects play a crucial role in its development (4). While risk factors like age, obesity, alcohol consumption, and estrogen exposure can contribute to the development of breast cancer, the presence of genetic factors and family history are considered the most influential risk factors for this disease (5). It is believed that breast cancer may arise from non-invasive lesions that are not specific to the breast, such as atypical ductal hyperplasia and ductal carcinoma in situ (DCIS). Ductal carcinoma in situ (DCIS) is a complex disease characterized by various clinical manifestations, histological subtypes, and biological behaviors (6). Over the past few decades, the prevalence of DCIS has witnessed an increase, likely attributed to improved screening programs. Currently, DCIS accounts for approximately 20-25% of all newly diagnosed breast cancers (7). While DCIS is recognized as a direct precursor to invasive carcinoma, the rate of progression can significantly vary, with certain types of DCIS exhibiting more rapid progression than others. In grading and assessing the prognosis for DCIS, conventional parameters such as histological patterns, nuclear grade, and the presence of necrosis are utilized. However, relying solely on morphological features does not fully capture the underlying biological characteristics of this disease. Therefore, it is crucial to identify markers that can effectively predict recurrence or progression to invasive carcinoma in DCIS (7, 8). In recent years, several molecular markers in breast cancer have become increasingly significant, serving as not only prognostic indicators but also predictors of treatment response. Among these markers are steroid receptors (ER/PR), Her2/neu, and Ki67(8). Considering that ductal carcinoma in situ is recognized as the direct precursor to invasive carcinoma, and taking into account the role of molecular markers, histological patterns, and nuclear grade in determining prognosis, treatment response, and recurrence rates, our study aims to examine the similarities or differences in these variables between the in situ and invasive components of carcinoma. Further investigations focused on ductal carcinoma in situ (DCIS) and invasive carcinoma of the breast are crucial for uncovering the still unknown aspects of this field. Thus, the present study aimed to contribute to this knowledge by comparing the structural, cytological, and immunohistochemical features of carcinoma in situ and the invasive component in women diagnosed with breast cancer.

## Material and Methods

**Fig. 1:**
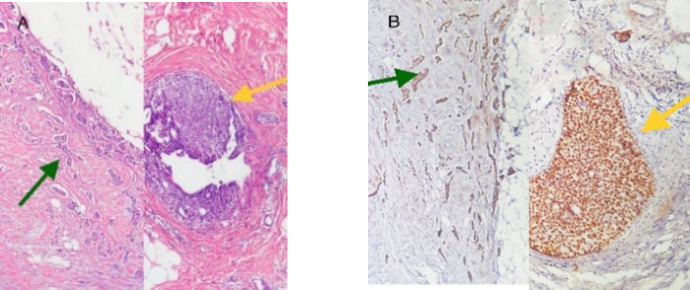
Invasive ductal carcinoma component (green arrow), ductal carcinoma in situ component (yellow arrow). A: Hematoxylin & Eosin; B: ER immunohistochemical staining

The present study was a descriptive-analytical and cross-sectional case study. The study population comprised all women with breast cancer who visited Al-Zahra Hospital in Isfahan City between 2015 and 2021 for the diagnosis of their disease. The inclusion criteria for the study were having invasive breast carcinoma, possessing a complete pathology file at Al-Zahra Hospital, being examined for immunohistochemistry (IHC) at Al-Zahra Hospital, and not receiving neoadjuvant treatment. The exclusion criteria involved samples that were included in the IHC slide but did not have in situ carcinoma. Sampling was conducted through a census of all eligible cases who visited Al-Zahra Hospital between 2015 and 2021. After examining 160 cases and applying the inclusion and exclusion criteria, a total of 51 cases of women with breast cancer were ultimately included in the study. The methodology involved obtaining permission from the research deputy, and subsequently reviewing all pathology reports related to breast cancer in women who visited Al-Zahra Hospital during the specified period. This encompassed biopsy samples as well as resection specimens. The cut-off for considering DCIS in biopsy samples was 2 mm. All samples underwent re-examination by a second pathologist. If the IHC analysis of these samples was conducted at the same centers, the corresponding IHC slides were retrieved from the pathology archive and reviewed by the pathologist. In cases where both in situ and invasive components were present in the IHC slides, the sample was included in the study (Figure 1). The pathologist recorded the structure, grade, and results of IHC markers for both the in situ and invasive components in a checklist.

In this study, the criteria utilized for classifying invasive and in situ breast carcinoma involved assessing the structure, grade, and IHC markers, including ER receptor, PR receptor, Ki-67, and Her-2. Breast invasive carcinoma and DCIS can be present as a spectrum, with low-grade (I), intermediate-grade (II), and high-grade (III) lesions, but for data analysis, each group is categorized into two levels: low (low) and high (intermediate & high) (9).

For ER and PR receptors, a 1% cut-off was applied. If the expression was less than 1%, it was considered negative, while more than 1% was considered positive. Ki-67 was evaluated in the hot spot and had a cut-off of 15%. If the expression was below 15%, it was categorized as low, whereas above 15% was classified as high. The Her2 degree is determined on a scale of 0 to 3, following the standard criteria. Grade zero is assigned to cases where the membrane shows no staining or less than 10% of the cells are stained. Grade 1 is given to cases where the membrane is focally stained in more than 10% of the cells. Grade 2 is designated for cases where the membrane is completely and weakly or moderately stained in more than 10% of the cells, and this is considered negative. Grade 3 is assigned to cases where the membrane is completely and intensely stained in more than 10% of the cells, and this is considered positive. Regarding nuclear grade, it encompasses three grades: 1, 2, and 3. Grade 1 represents low-grade, while grades 2 and 3 signify high-grade (10). The structural patterns assessed in this study include NOS (invasive), Solid (in situ), Cribriform, Medullary, Comedo, Papillary, Micropapillary, Mucinous, and LCIS. For data collection, two checklists were utilized, one for in situ samples and another for invasive samples, each containing 5 items. Qualitative data were analyzed using frequency/percentage, while quantitative data were analyzed using mean/standard deviation. To compare the data between the in situ component and the invasive component, the Chi-square test and SPSS20 software were employed. Additionally, for data analysis, the chi-square test and McNemar's test were utilized.

## Results

A total of 51 patients aged 27 to 74 years with a mean age of 48.78±11.19 years were examined. The largest proportion of the patients was in the age group of 51-60 years (35.3%), while the smallest proportion was found in older than 60 years (11.8%). Among the 51 samples of carcinoma in situ, 38 cases (74.5%) were classified as high-grade. Additionally, among the 51 samples of invasive carcinoma, 40 cases (78.4%) were also categorized as high-grade (Table 1). 

Based on the results presented in Tables 2, 3, and 4, it was observed that among the 51 examined patients, 36 cases (70.6%) had both carcinoma in situ and invasive carcinoma graded as "high". Overall, the grade of in situ and invasive carcinoma was the same in 45 patients, accounting for 88.2% of the cases. In contrast, in 6 patients, the grades of in situ and invasive carcinoma differed, representing 11.8% of the cases. The result of McNemar's test indicated that there was no significant difference between the grade types of in situ and invasive carcinomas (*P*=0.687) (Table 5).

In the carcinoma in situ group, the largest proportion was of the solid structure type, accounting for 45.1% of the cases. The cribriform structure was the second most common, representing 23.5% of the cases, followed by comedo with a frequency of 17.6%. In the group of carcinomas with an invasive component, the NOS structure type accounted for the largest proportion, comprising 90.2% of the cases. Based on the results presented in Table 2, it was observed that among the 51 examined patients, 23 cases (45.1%) had a solid structure in situ carcinoma and NOS structure in invasive carcinoma. In 23 patients (45.1%), the invasive carcinoma exhibited the NOS structure, while the in-situ carcinoma had structures other than solid. In general, the structure of in situ and invasive carcinoma was the same in 23 patients (45.1%) and different in 28 patients (54.9%). The result of the chi-square test indicated that there was no significant difference between the number of cases with the same and different structures of in situ and invasive carcinomas (*P*=0.484) (Table 2).Among 51 samples of carcinoma in situ, ER.PR value was found negative in 12 samples (23.5%) and positive in 39 samples (76.5%). In invasive carcinoma samples, ER.PR value was found to be negative in 11 samples (21.6%) and positive in 40 samples (78.4%) (Table 6).

**Table 1:** Frequency distribution and descriptive indicators of the patient's age

Variable	Category	Frequently distribution		Descriptive index			
Age		N	%	The least	The most	M	SD
	<40 years	12	23.5	27.00	74.00	48.78	11.19
	41-50 years	15	29.4				
	51-60 years	18	35.3				
	> 60 years	6	11.8				
	Total	51	100				

**Table 2:** Comparison of the frequency distribution of the immunohistochemical features (ER.PR) of carcinoma in situ and invasive components of the breast

In situ invasive		Negative		Positive		Total		P*
		N	%	N	%	N	%	
Negative		10	19.6	2	3.9	12	23.5	1.000
Positive		1	2.0	38	74.5	39	76.7	
Total		11	21.6	40	78.4	51	100.0	

*Calculated based on the McNemar test

**Table 3:** Comparison of frequency distribution of the immunohistochemical features (Ki67) of carcinoma in situ and invasive components of the breast

In situ invasive	Low		High		Total		P*
	N	%	N	%	N	%	
Low	20	45.5	2	4.5	22	50.0	0.180
High	7	15.9	15	34.1	22	50.0	
Total	27	61.4	17	38.6	44	100.0	

*Calculated based on the McNemar test

**Table 4:** Comparison of frequency distribution of the immunohistochemical characteristics (Her2) of carcinoma in situ and invasive components of the breast

In situ invasive	Negative		Positive		Unknown		Total							P*
	N	%	N	%	N	%	N			%			0.532	
Negative	19	42.2	1	2.2	1	2.2	21		46.7					
Positive	0	0.0	22	24.4	2	4.4	13	28.9						
Unknown	3	6.7	3	6.7	5	11.1	11	24.1						
Total	22	48.9	15	33.3	8	17.8	45	100.0						

*Calculated based on the McNemar-Booker test*

**Table 5:** Comparison of frequency distribution of the cytological characteristics of carcinoma in situ and invasive components part of the breast

In situ invasive	Low		High		Total		P*
	N	%	N	%	N	%	
Low	9	17.6	2	3.9	11	21.6	0.687
High	4	7.8	36	70.6	40	78.4	
Total	13	25.5	38	74.5	51	100	

**Table 6:** Comparison of frequency distribution of the structural characteristics of carcinoma in situ and invasive components of the breast

n situ invasive	Solid		other		Total		P*
	N	%	N	%	N	%	
NOS	23	45.1	23	45.1	46	90.0	0.484
Other	0	0.0	5	9.8	5	9.8	
Total	23	45.1	28	54.9	51	100.0	

*Calculated based on chi-square test

Based on the results presented in Table 2, it was observed that in 38 samples, accounting for 74.5% of both in situ and invasive carcinomas, the ER.PR value was positive. Additionally, in 10 samples, representing 19.6% of both in situ and invasive carcinomas, the ER.PR values were negative. Therefore, in general, the value of ER.PR of in situ and invasive carcinoma was the same in 48 samples (94.1%) and different in 3 samples (5.9%). The result of McNemar's test did not reveal a significant difference between ER.PR type of in situ and invasive carcinomas (*P*=1.00) (Table 2).

Among the 51 samples of carcinoma in situ, the amount of Ki67 was recorded in 44 samples and its value was low in 27 samples (61.4%) and high in 17 samples (38.6%). Among 51 samples of invasive carcinoma, the amount of Ki67 was recorded in 44 samples, the amount of Ki67 was low in 22 samples (0.50%) and high in 22 samples (0.50%). Based on the results presented in Table 3, it was found that Ki67 levels were recorded in both in situ and invasive carcinomas in a total of 44 samples. Among these samples, in 20 cases (45.5%), both in situ and invasive carcinomas had low Ki67 levels, while in 15 samples (34.1%), both in situ and invasive carcinomas had high Ki67 levels. Therefore, in general, the amount of Ki67 of in situ and invasive carcinoma was the same in 35 samples (79.6%) and different in 9 samples (20.4%). The result of McNemar's test indicated that there was no significant difference between the Ki67 types of in situ and invasive carcinomas (*P*=0.180) (Table 3).

Among the 51 samples of carcinoma in situ, the Her2 level was recorded in 47 samples. Out of these 47 samples, the amount of Her2 was negative in 23 samples (48.9%), positive in 15 samples (31.9%) and equivalent in 9 samples (19.1%). Among the 51 samples of invasive carcinoma, the Her2 level was recorded in 45 samples. Out of these 45 samples, the Her2 value was negative in 21 samples (46.7%), positive in 13 samples (28.9%) and equivalent in 11 samples (24.4%). Based on the results presented in Table 4, it was found that Her2 levels were recorded in both in situ and invasive types of carcinomas in a total of 45 samples. Among these samples, in 19 cases (42.2%), both in situ and invasive carcinomas were Her2 negative. In general, the amount of Her2 in situ and invasive carcinoma was the same in 35 samples (77.8%) and different in 10 samples (22.2%) (Table 4).

## Discussion

Breast carcinoma is the most common malignancy among women and one of the leading causes of death worldwide (11). It is believed that breast cancer originates from non-invasive lesions such as atypical ductal hyperplasia (ADH) and ductal carcinoma in situ (DCIS) (12). While the exact mechanisms by which invasive breast cancer develops from these lesions are not yet fully understood, there is growing evidence suggesting multiple pathways of progression to invasive breast cancer based on histology and degree of differentiation (13). The role of molecular markers in breast cancer goes beyond being prognostic indicators; they are also predictive of treatment response. Various markers, including estrogen receptor (ER) and progesterone receptor (PR) (14), Her2/neu (15), and Ki67(16), have gained significant attention. However, the expression of these markers in ductal carcinoma in situ (DCIS) and invasive ductal carcinoma (IDC) has not been thoroughly evaluated. This study aimed to investigate the differences in structure, grade, and tumor markers between the invasive and in situ components of breast carcinoma in patients presenting with both components simultaneously. Our findings indicated that the phenotype of IDC closely resembled, but not completely that of the in-situ component.

This study's findings on the age of prevalence align with similar studies, indicating consistent results. The age range and the highest age of involvement observed in this study are comparable to those reported in other studies (1). Nuclear grade is indeed a significant prognostic factor regarding the recurrence and progression of malignancy. Goldstein* et al.*'s study demonstrated that younger age in patients is a risk factor for local recurrence, which was associated with higher nuclear grade (17). In the current study, the majority of patients fell within the 51-60 age range and exhibited a high nuclear grade, suggesting that progression toward invasion is linked to a higher grade. Notably, nuclear grade holds greater importance than the structural pattern, as supported by previous studies (18). Thus, nuclear grade is regarded as a fundamental method for assessing biological invasion. It serves as an indicator of the probability of invasion, with higher nuclear grades in the in-situ component correlating with an increased likelihood of invasion (19). In the study of Saroud* et al.*, the grade was consistent in both in situ and invasive components, and the majority of inconsistent cases were observed in the upper grade of the invasive component (20). Similarly, in our study, when there was a discrepancy in the grade of the two components, the more aggressive component tended to have a higher grade. This finding suggests the possibility of a statistically significant relationship, particularly when considering a larger sample size.

DCIS can exhibit various structural patterns, including solid, cribriform, micropapillary, papillary, and uncommon forms such as apocrine, signet ring, neuroendocrine, spindled, squamous, or clear cells (21). While certain combinations of nuclear grades and structural patterns may occur more frequently, it is important to note that any combination is possible (22). Previous research has indicated that high nuclear grades are more commonly associated with the solid type of DCIS (23). Solid DCIS is characterized by the proliferation of neoplastic cells, leading to the filling, expansion, and deviation of the duct. In solid DCIS, the nuclear grade can vary from high to low, although low grades are relatively uncommon in this subtype. It is worth noting that the presence of a low nuclear grade combined with a solid structure can sometimes lead to a diagnostic challenge, as it may resemble lobular carcinoma in situ (21, 24). In this study, in the in situ and invasive components, the most common structural patterns were solid and NOS patterns respectively, and these two structures were considered equivalent to each other. Few studies have compared the structure in both invasive and in situ components of breast cancer. The matching of solid structure and NOS in two groups is a significant result that can be used in morphometric studies.

Steroid receptors play a crucial role not only in determining the phenotype as a prognostic factor but also in predicting the response to hormone therapy (14). In most studies, there is concordance between the hormone receptor status of the in situ and invasive components (16). In the study conducted by Steinman* et al.*, discrepancies between ER and PR statuses in the invasive and in situ components were predominantly observed in high-grade tumors. These discrepancies often manifested as positive values in the in-situ component and negative values in the invasive component (25). In the study conducted by Yanbiao Liu* et al.*, it was found that both the in situ and invasive components exhibited high and similar levels of ER and PR. However, in the study by Yu* et al.*, lower levels of ER were reported in invasive and microinvasive lesions. The researchers speculated that ER-negative cancer cells might play a more significant role in primary invasion, which contradicts the findings of the study by Yanbiao Liu* et al. *(26). In our study, the majority of the samples showed consistent levels of ER and PR expression in both the in situ and invasive components. However, there were three cases where a discrepancy was observed between the invasive and in situ components. This finding suggests the need for further studies from histological and biological perspectives to better understand and clarify this situation.

Ki67 is utilized as a prognostic marker in breast cancer patients. Furthermore, it is considered a predictive marker for chemotherapy response. Ki67 expression has not been observed in both the in situ and invasive components (27). In the study conducted by Clark* et al.*, the proliferative index of Ki67 was lower in the in-situ component compared to the invasive component (28). However, it has been observed that the Ki67 index increases during the progression of the in-situ component to the invasive side. This increase in the Ki67 index is believed to be associated with its specific role in tumorigenesis (29). In our study, it was found that the levels of Ki67 were comparable between the in-situ component and the invasive component in most samples. However, in certain cases, although not statistically significant, the in-situ component exhibited a lower proliferative index compared to the invasive component. This observation aligns with the findings of Clark's study. 

Her2 is a growth factor receptor that typically does not exhibit high expression in benign and normal breast lesions (30). In a recent study, it was concluded that high expression of Her2 in DCIS is the only significant factor predicting invasion (31). Roses* et al.* demonstrated that high nuclear grade, large lesion size, and high expression of Her2 are associated with disease invasion (32). Previous studies suggest that during the progression from ADH to DCIS, the Her2-positive phenotype is acquired, and during the progression to IDC, it may be eliminated by immune responses (33). On the other hand, the study conducted by Wan* et al.* demonstrated that high expression of Her2 in DCIS was associated with microinvasion (34). In the study conducted by Mylonas* et al.*, it was found that the levels of Her2 were comparable between the in situ and invasive components of the same tumor. This finding supports previous results that have reported similar observations (35,36,37). In the present study, it was observed that the levels of Her2 in both the in situ and invasive components of most samples were similar and negative. Considering that the majority of the samples had a high nuclear grade, it can be inferred that high expression of Her2 is not commonly observed in the invasions following the in-situ component. In this study, the majority of the mismatch cases showed either Her2 positivity or negativity in the in-situ component, while the Her2 status in the invasive component remained unclear. It is important to note that the standard method for examining Her2 is the FISH method, which can provide clearer and more accurate results when applied to a larger sample size.

## Conclusion

In general, it can be concluded that in breast carcinoma, in situ and invasive components may not exhibit significant differences in terms of structural features, nuclear grade, and other immunohistochemical markers. This finding can contribute to the understanding of the pathogenesis of invasive breast cancer, and the degree of inconsistency observed in each of the examined indicators can serve as an indirect factor for evaluating the extent of tumor heterogeneity. Differences between in situ and invasive biomarker expression, although not significant, show how in situ components can’t be representative of invasive components for treatment choices. It is likely that with a larger sample size, the results would be more reliable, and therefore, studies with a higher sample size in this field are recommended. 
